# Development of a multimodel predictive framework for gangrenous cholecystitis with SHAP-based interpretability

**DOI:** 10.1186/s12911-026-03705-9

**Published:** 2026-10-27

**Authors:** Qi-Guang Niu, Yang Yu, Fang Xu, Wei-Qi Kong, Jia-Yang Li, De-Feng Tang, Yi Wang

**Affiliations:** 1 Department of General Surgery, Tong Ren Hospital, Shanghai Jiao Tong University School of Medicine, No. 1111 XianXia Road, Changning District, Shanghai City, 200336 China; 2 Nutritional Department, Tong Ren Hospital, Shanghai Jiao Tong University School of Medicine, Shanghai, 200336 China

**Keywords:** Gangrenous cholecystitis, Machine learning, Preoperative prediction, SHAP

## Abstract

**Background:**

Gangrenous Cholecystitis (GC) is a critical pathological type of acute cholecystitis that tends to induce gallbladder wall necrosis or perforation, thereby leading to localized or diffuse peritonitis and is associated with high morbidity and mortality. Due to its nonspecific clinical symptoms and strong heterogeneous manifestations, timely and accurate identification of GC preoperatively remains a clinical challenge. To address this unmet clinical need, we developed an interpretable machine learning (ML) model using routinely available preoperative variables to facilitate the early prediction of GC and support clinical decision-making.

**Methods:**

This was a retrospective cohort study, including 487 patients with acute cholecystitis who underwent laparoscopic cholecystectomy in our hospital between February 2020 and July 2023. After initial univariate analysis and logistic regression, nine ML models were constructed, including logistic regression (LR), random forest (RF), XGBoost, LightGBM, support vector machine (SVM), artificial neural network (ANN), k-nearest neighbor (KNN), linear discriminant analysis (LDA), and a Stacking ensemble model. LASSO regression was used to select key preoperative predictors. Model performance was evaluated using area under the receiver operating characteristic curve (AUC), accuracy, F1-score, sensitivity, specificity, decision curve analysis (DCA), and calibration curves. SHAP (SHapley Additive exPlanations) analysis was performed to enhance model interpretability.

**Results:**

LASSO regression identified five key preoperative predictors: preoperative C-reactive protein (pre-CRP), gallbladder diameter, pericholecystic exudation, albumin (ALB), and basophil percentage (BA). Among all constructed models, XGBoost and RF exhibited superior predictive performance, with AUC values of 0.90 and 0.89, respectively. Both models demonstrated robust discrimination, good calibration, and significant net clinical benefit across the entire range of decision thresholds. SHAP analysis revealed that pre-CRP and gallbladder diameter were the most influential predictors of GC, while BA and ALB showed inverse or sample-dependent associations with GC occurrence.

**Conclusions:**

We developed an interpretable, non-invasive, and high-performance ML framework for preoperative GC prediction using routine clinical variables. This framework provides a valuable clinical tool to assist surgeons in decision-making and early risk stratification of patients with suspected GC, potentially improving patient outcomes by enabling timely intervention.

## Introduction

Gangrenous cholecystitis (GC) is a critical pathological subtype of acute cholecystitis, pathologically characterized by progressive ischemia, necrosis, and even perforation of the gallbladder wall [1, 2]. It is often accompanied by systemic inflammatory response syndrome or sepsis, with rapid disease progression and poor prognosis. Without timely surgical intervention, it can readily lead to serious consequences such as gallbladder perforation, intra-abdominal infection, and septic shock [3, 4].The mortality rate of gangrenous cholecystitis is significantly higher than that of uncomplicated acute cholecystitis. Although GC accounts for approximately 10%–40% of all acute cholecystitis cases, its preoperative diagnostic rate remains below 10%, and the diagnosis mostly relies on intraoperative findings or postoperative pathological examination [1, 5]. Such diagnostic delay not only results in the loss of the optimal treatment window, but also markedly increases surgical difficulty and the risk of postoperative complications [3, 6].Therefore, establishing an auxiliary tool that can accurately predict the risk of gangrenous cholecystitis preoperatively is of great clinical significance for optimizing the timing of intervention, reducing unnecessary delays, and improving patient prognosis.

Most current clinical predictions of GC rely on the combined determination of routine blood tests, imaging, and individual biochemical indicators, or risk assessment, by constructing traditional multifactor-based logistic regression models [4, 5, 7–9]. However, the predictive power of logistic regression is limited when dealing with high-dimensional, non-linear feature interactions. Its stability and generalizability are often compromised when applied to real-world clinical datasets characterized by imbalanced classes or skewed feature distributions [8, 10]. Furthermore, the performance of certain indicators varies significantly across patient populations, further undermining model reliability [11, 12]. Although some studies have attempted to construct GC risk scoring systems or predictive factor combinations, most suffer from limited sample sizes, lack standardized modeling processes, and often lack external validation [7].

In recent years, the accumulation of medical data and advances in computational power have spurred the application of machine learning in clinical prediction [12]. Compared to traditional regression models, machine learning algorithms such as random forests, gradient boosting decision trees, and support vector machines can better capture complex non-linear relationships among variables, offering superior performance in disease classification tasks [8, 13, 14]. Nevertheless, these high-performance models often sacrifice interpretability, making them difficult for clinicians to understand and trust, thereby limiting their adoption in real-world clinical settings. Thus, achieving a balance between predictive accuracy and model transparency remains a critical challenge in medical artificial intelligence.

This study aims to develop a preoperative prediction model for gangrenous cholecystitis that is both high-performing and interpretable, using multimodal clinical data. By applying ensemble learning algorithms such as XGBoost and random forests to model a refined set of core variables, and integrating the SHAP (SHapley Additive exPlanations) method to visualize and interpret model decision-making processes, we identify key features associated with GC risk. The resulting model not only improves predictive accuracy but also provides clinicians with a traceable and interpretable decision-support tool, potentially facilitating early diagnosis and timely intervention in gangrenous cholecystitis.

## Materials and methods

### Study samples

This was a single-center retrospective cohort study conducted at Tongren Hospital, Shanghai Jiao Tong University School of Medicine. Study data were retrieved from all patients who underwent cholecystectomy between February 2020 and July 2023. Comprehensive clinical data were collected from the Department of Hepatobiliary Surgery, including preoperative clinical characteristics, laboratory examinations, imaging evaluations, operative records, and postoperative pathological diagnoses.

Preoperative clinical features comprised age, sex, maximum preoperative body temperature, duration of disease onset, comorbidities, and peritonitis signs. Peritonitis was scored from 0 to 3, with 1 point assigned for tenderness, 1 point for rebound tenderness, and 1 point for muscular guarding, respectively. Laboratory test results included routine blood tests, coagulation profiles, procalcitonin levels, and biochemical indicators. With respect to imaging assessments, both abdominal ultrasound and computed tomography (CT) examinations were reviewed. The maximum longitudinal diameter (cm), maximum transverse diameter (cm), and wall thickness (mm) of the gallbladder, as well as the presence or absence of pericholecystic effusion (0 = no, 1 = yes) and gallstone incarceration (0 = no, 1 = yes), were independently measured by a radiologist with more than 5 years of clinical experience, who was blinded to patient grouping and the study protocol.Intraoperative parameters extracted from surgical records included operation time (minutes), intraoperative blood loss (milliliters), severity of pericholecystic exudation, and the presence of gallbladder wall gangrene or perforation. After surgical resection, the gallbladder was dissected to evaluate the maximum diameter of gallstones (cm) and the presence of stone incarceration (0 = no, 1 = yes). All findings were further confirmed by final postoperative pathological diagnosis.

All ultrasound or computed tomography (CT) examinations and laboratory tests for all patients were performed and obtained within 48 h preoperatively, and all surgical procedures were completed within 48 h after hospital admission. Patients who underwent percutaneous transhepatic gallbladder drainage (PTGBD) were excluded from the study, as pathological specimens could not be obtained for these individuals.This patient population generally presents with complex clinical conditions: some are intolerant to general anesthesia, some refuse general anesthesia, and others may have already developed gallbladder wall gangrene or perforation, leading to unstable vital signs or even critical illness. Given that the inclusion of such patients may introduce substantial selection bias, they were excluded from the final analysis.

According to the diagnostic criteria and classification of acute cholecystitis outlined in the Tokyo Guidelines 2018 (TG18), the following inclusion and exclusion criteria were established:

Inclusion criteria:

1. Age ≥ 18 years;
2. Fulfillment of the TG18 clinical diagnostic criteria for acute cholecystitis, including right upper quadrant tenderness, systemic inflammatory response, and imaging evidence of gallbladder disease;
3. Underwent cholecystectomy at the study center, with postoperative pathological confirmation of acute or gangrenous cholecystitis;
4. Availability of at least one preoperative hematological test and one imaging examination;
5. Complete clinical data sufficient for model development and analysis.

Exclusion criteria:

1. Cholecystectomy performed as an adjunct to other abdominal surgeries rather than as a primary indication for cholecystitis;
2. Patients with gallbladder carcinoma or those undergoing surgery for other malignancies;
3. Patients who underwent elective surgery following percutaneous transhepatic gallbladder drainage or other biliary interventions;
4. Patients with concomitant severe hepatic dysfunction, pancreatitis, or acute cholangitis;
5. Incomplete clinical data preventing variable extraction or outcome determination.

Based on the above criteria, a total of *N* = 487 patients who underwent cholecystectomy were included in the study. Among them, 94 cases (19.3%) were pathologically diagnosed as gangrenous cholecystitis (GC), while the remaining 393 cases (80.7%) were classified as non-GC. All data were collected prior to surgery, and both laboratory and imaging results were obtained within 48 h before surgery to ensure the predictive validity of the model in a prospective context.

### Variable selection and preprocessing

Prior to model construction, the raw dataset underwent cleaning and normalization procedures. Features with a missing rate greater than 20% were excluded. For continuous variables with partial missing values, multiple imputation techniques were applied to fill in the gaps. To minimize the impact of differing scales on modeling stability, all continuous variables were standardized using Z-score normalization.

In the initial stage of variable selection, independent two-sample t-tests were employed to compare preoperative clinical characteristics between the GC and non-GC groups. Variables showing statistically significant differences between the two groups (*P* < 0.05) were retained for further consideration. To identify features with potential clinical predictive value, univariate logistic regression analysis was performed on the candidate variables, quantifying their risk effects by extracting odds ratios (ORs) along with 95% confidence intervals.

### Feature selection and model construction

Following preliminary screening, to further reduce dimensionality and enhance the generalizability of the model, we employed the Least Absolute Shrinkage and Selection Operator (LASSO) regression method for feature compression and selection. By incorporating an L1 regularization term, LASSO enables automatic variable selection, effectively handling multicollinearity and mitigating overfitting risks.

Subsequently, nine classification models were developed, including logistic regression, random forest, XGBoost, LightGBM, and support vector machine (SVM), among others. Model performance was comprehensively evaluated based on accuracy, F1-score, recall, and the area under the receiver operating characteristic curve (AUC). In addition, the two best-performing models—XGBoost and random forest—were subjected to SHAP (SHapley Additive exPlanations) analysis to enhance model interpretability by visualizing and quantifying feature contributions to individual predictions. All machine learning models were implemented using Python (version 3.12.4). For each model, grid search combined with 5-fold cross-validation was used to determine optimal hyperparameters. The training-to-testing set split was maintained at an 80:20 ratio, and the testing set was held out and never used during model training or tuning. All statistical analyses, including t-tests and univariate logistic regression, were conducted using Python. A two-sided P-value < 0.05 was considered statistically significant.

### Interpretability analysis

To comprehensively evaluate the performance of the constructed models in preoperative prediction of gangrenous cholecystitis, the dataset was divided into training and testing sets in an 80:20 ratio. Five-fold cross-validation was applied within the training set to optimize model hyperparameters. A total of nine mainstream classification models were developed, including logistic regression, random forest, extreme gradient boosting (XGBoost), LightGBM, support vector machine (SVM), k-nearest neighbors (KNN), linear discriminant analysis (LDA), artificial neural network (ANN), and a Stacking ensemble model.

To enhance the clinical applicability and transparency of the predictive models, the SHAP (SHapley Additive exPlanations) interpretability framework was employed. SHAP analysis was performed on the two best-performing model to retrospectively quantify the contribution of each feature to the model outputs. Based on Shapley values from cooperative game theory, SHAP assigns an additive importance score to each feature for every individual prediction, thereby enabling a quantitative and interpretable explanation of the decision-making logic. SHAP analysis was applied to the final XGBoost and random forest models using the TreeExplainer algorithm. SHAP summary plots were generated to visualize global feature importance and directionality. The SHAP values for individual features were used to assess their contribution to the model’s output across all patients.

### Performance evaluation metrics

Model performance was evaluated using multiple metrics, including accuracy, precision, recall (sensitivity), F1-score, area under the receiver operating characteristic curve (AUC), calibration curves, and decision curve analysis (DCA). The F1-score was defined as the harmonic mean of precision and recall, balancing false positives and false negatives. The AUC was used to measure overall classification performance. Calibration was assessed by comparing predicted probabilities against observed outcomes. Clinical utility was further evaluated using decision curve analysis and clinical impact curves across varying threshold probabilities.

## Results

### Baseline characteristics

Among the 487 patients who underwent cholecystectomy, 94 (19.3%) were pathologically confirmed to have gangrenous cholecystitis (GC), while the remaining 393 (80.7%) were classified as non-GC cases. To explore preoperative differences between the two groups, independent t-tests were conducted on all continuous variables. Variables with statistically significant differences (*P* < 0.05) are summarized in Table 1. The results revealed that preoperative C-reactive protein (CRP) levels were significantly higher in the GC group compared to the non-GC group (151.73 ± 100.54 mg/L vs. 57.82 ± 76.42 mg/L, *P* < 0.001), indicating a more pronounced systemic inflammatory response. Gallbladder diameter was also significantly greater in the GC group (5.73 ± 1.59 cm vs. 4.57 ± 1.20 cm, *P* < 0.001), reflecting increased intraluminal pressure or bile retention. The presence of pericholecystic effusion, a key radiologic indicator of acute inflammation, was markedly more common in GC patients (0.80 ± 0.40 vs. 0.38 ± 0.49, *P* < 0.001), suggesting potential gallbladder wall necrosis or surrounding fluid accumulation. Moreover, serum albumin (ALB) levels were significantly lower in the GC group (42.58 ± 10.17 g/L vs. 46.54 ± 9.92 g/L, *P* = 0.057), potentially reflecting malnutrition or protein loss associated with systemic inflammation.

Several additional laboratory indicators demonstrated statistically significant differences between the GC and non-GC groups, suggesting their potential relevance in disease pathophysiology and prognostication. Specifically, the percentage of basophils (BA) was markedly lower in GC patients, which may reflect altered immune response or allergic signaling suppression during severe inflammation. Serum γ-glutamyltransferase (γGT) levels, an indicator of hepatobiliary stress, were reduced in the GC group, possibly due to gallbladder wall necrosis impairing bile flow. Moreover, prolonged prothrombin time-international normalized ratio (PT-INR) and activated partial thromboplastin time (APTT) suggest a tendency toward coagulopathy or systemic inflammatory-mediated coagulatory dysregulation. Additionally, lower levels of alanine aminotransferase (ALT) may indicate diminished hepatocellular injury in GC relative to other forms of cholecystitis. Collectively, these findings not only reinforce the systemic impact of GC but also contribute valuable candidates for subsequent feature selection and model construction.

**Table 1:** Comparison of preoperative clinical characteristics between GC and Non-GC patients

Variable	Group (mean ± SD)		t-value	*P*-value
	Non-GC (*n* = 393)	GC (*n* = 94)		
Age (years)	60.49 ± 15.18	63.96 ± 14.09	-2.019	0.044*
CRP (mg/L)	57.82 ± 76.42	151.73 ± 100.54	-8.488	< 0.001**
Neutrophils (%)	82.46 ± 9.81	86.87 ± 5.78	-5.686	< 0.001**
Lymphocyte/WBC ratio	1.36 ± 1.61	0.63 ± 0.99	5.589	< 0.001**
Lymphocytes (%)	11.80 ± 8.46	7.90 ± 7.77	4.305	< 0.001**
Monocytes (%)	5.07 ± 2.11	5.61 ± 2.13	-2.213	0.027*
Eosinophils (%)	0.57 ± 0.99	0.22 ± 0.44	5.236	< 0.001**
Basophils (%)	0.16 ± 0.18	0.07 ± 0.08	7.983	< 0.001**
Prothrombin Time (s)	12.11 ± 1.41	13.19 ± 2.45	-4.127	< 0.001**
PT-INR	1.02 ± 0.13	1.12 ± 0.21	-4.516	< 0.001**
Glucose (mmol/L)	7.42 ± 2.63	8.85 ± 3.45	-3.769	< 0.001**
Thrombin Time (s)	15.26 ± 6.26	13.92 ± 2.67	2.041	0.042*
Fibrinogen (g/L)	4.01 ± 1.55	5.33 ± 1.55	-7.431	< 0.001**
ALT (U/L)	71.74 ± 126.76	48.43 ± 84.99	2.148	0.033*
γ-GT (U/L)	100.54 ± 156.34	69.10 ± 78.38	2.784	0.006**
Body temperature (°C)	37.48 ± 0.81	37.81 ± 0.83	-3.575	< 0.001**
Gallbladder length (cm)	9.48 ± 1.71	10.61 ± 1.96	-5.146	< 0.001**
Gallbladder diameter (cm)	4.57 ± 1.20	5.73 ± 1.59	-6.644	< 0.001**
Wall thickness (mm)	5.70 ± 2.53	6.77 ± 2.73	-3.615	< 0.001**
Pericholecystic effusion	0.38 ± 0.49	0.80 ± 0.40	-8.555	< 0.001**
Diabetes mellitus (%)	0.16 ± 0.37	0.31 ± 0.46	-2.834	0.005**
Hypertension (%)	0.35 ± 0.48	0.51 ± 0.50	-2.790	0.006**
Peritoneal Inflammation Score				0.221
0	6(1.5%)	0(0.0%)		
1	248(63.1%)	62(66.0%)		
2	130(33.1%)	27(28.7%)		
3	9(2.3%)	5(5.3%)		

*P < 0.05, **P < 0.01. Data are presented as mean ± standard deviation or proportions as appropriate. GC: gangrenous cholecystitis; ALT: alanine aminotransferase; γ-GT: gamma-glutamyltransferase; PT-INR: prothrombin time-international normalized ratio

### Feature selection via LASSO regression

**Fig. 1:**
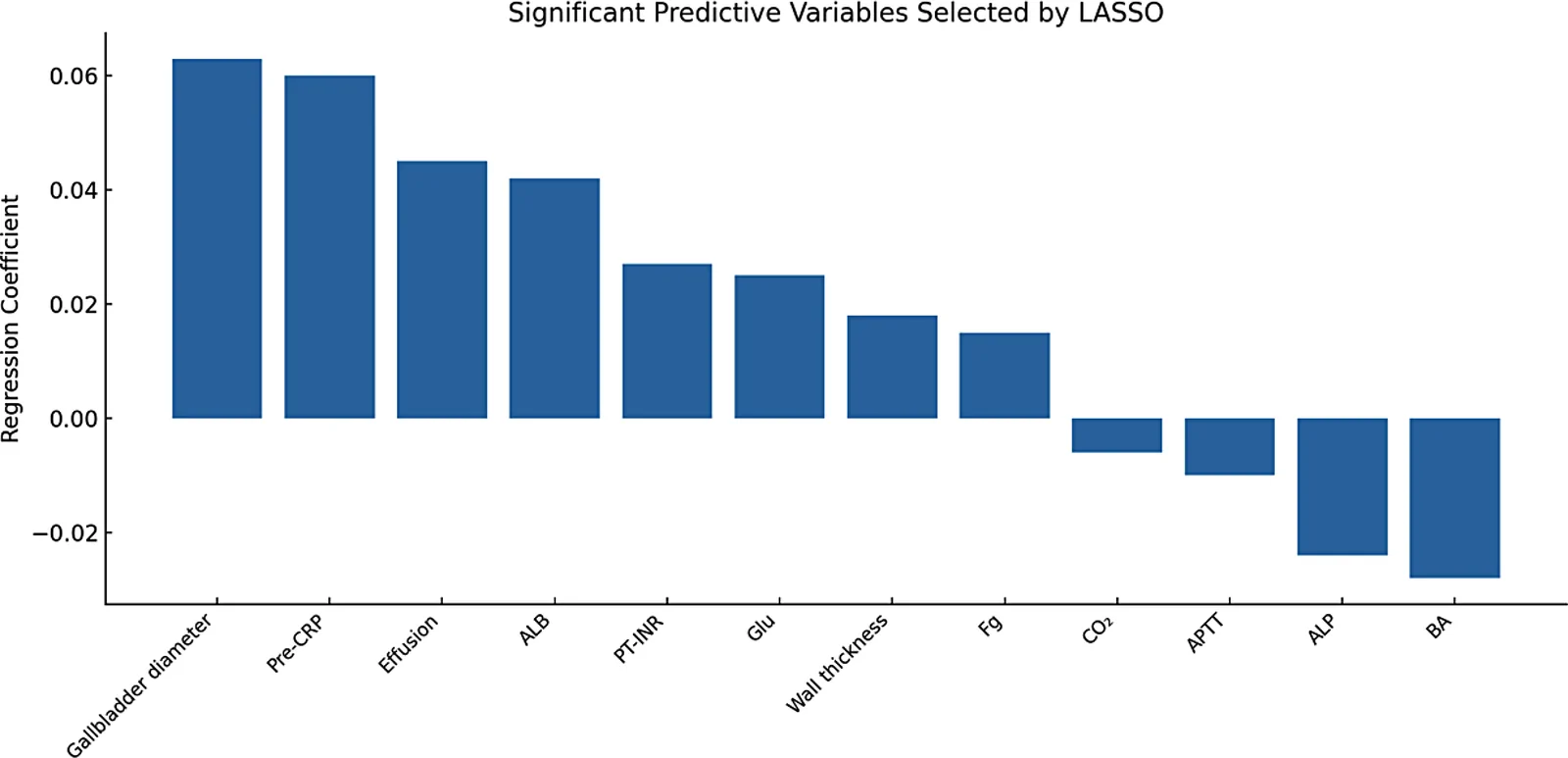
Significant variables screened by LASSO regression analysis and distribution of their regression coefficients. The figure shows the standardised regression coefficients of each variable in the LASSO regression model. Variables with positive coefficients, such as gallbladder diameter, pre-CRP, and exudation, make a positive contribution to the model output, while variables with negative coefficients make a negative contribution

To enhance the robustness of feature selection and mitigate the potential impact of multicollinearity on model performance, we applied the Least Absolute Shrinkage and Selection Operator (LASSO) regression following initial univariate analysis and logistic regression screening. As shown in Fig. 1, LASSO identified a subset of variables with non-zero regression coefficients that contributed meaningfully to the model’s predictive capacity. Among the positively weighted variables, gallbladder diameter, preoperative C-reactive protein (pre-CRP), and presence of pericholecystic effusion demonstrated the highest coefficients, indicating a strong positive association with gangrenous cholecystitis risk. These variables reflect core pathophysiological processes, specifically, gallbladder distension, systemic inflammation, and localized fluid accumulation, all of which are hallmark features of GC progression. In addition, serum albumin (ALB) and basophil percentage (BA) also displayed notable contributions. ALB, a negative acute-phase protein, likely reflects the catabolic and inflammatory burden in GC patients, while decreased BA levels may indicate immune dysregulation or altered allergic responses under severe inflammation. Interestingly, although BA exhibited a negative coefficient in LASSO, its magnitude remained substantial, suggesting that lower BA may serve as a discriminative feature in this context. By contrast, other variables such as PT-INR, glucose, ALP, and APTT, despite showing group-level significance in earlier analyses, received relatively small or near-zero coefficients in the LASSO model. This suggests their limited independent predictive contribution after penalization for redundancy and intercorrelation.

It is worth noting that some features with negative coefficients (e.g., ALP, Fg, CO₂) were not entirely excluded but were retained with minimal weights, implying marginal or context-specific effects. However, to optimize model simplicity and generalizability, we ultimately selected the five most influential features, pre-CRP, gallbladder diameter, pericholecystic effusion, ALB, and BA, for subsequent machine learning model construction and evaluation.

### Logistic regression modeling based on LASSO-selected variables

**Fig. 2:**
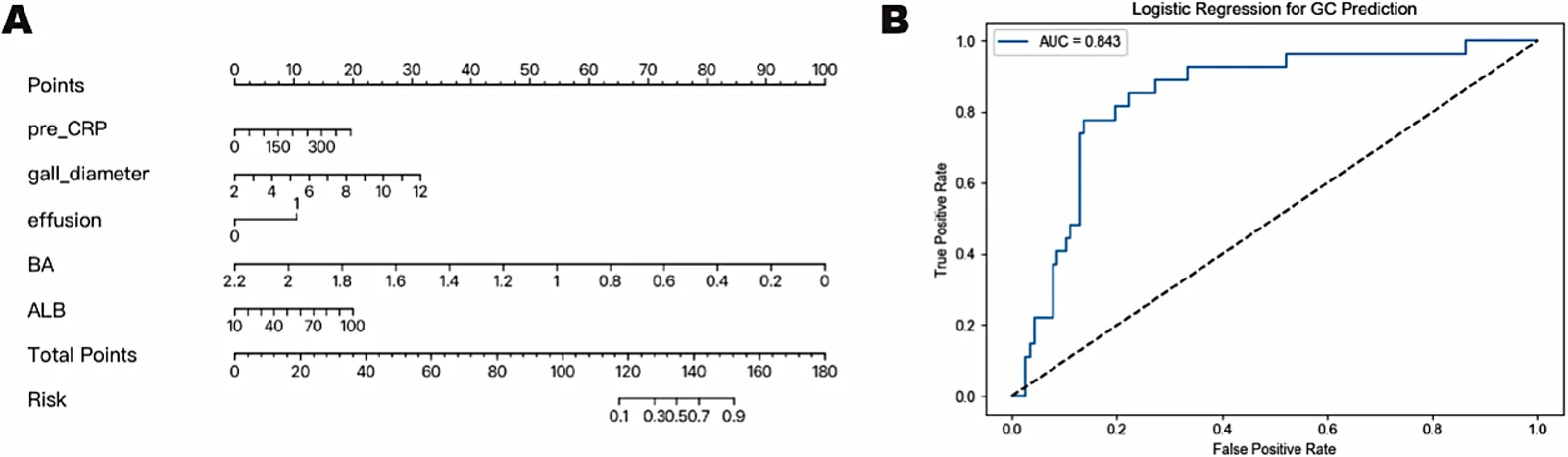
Prediction results of the logistic regression model. **A**. Nomogram for predicting gangrenous cholecystitis constructed using the logistic regression model. The nomogram is based on CRP, gallbladder diameter, exudation, BA, and ALB.The upper axis represents the scores (Points) corresponding to each variable, the middle axis represents the range of each variable, and the lower axis represents the mapping relationship between the total score and the corresponding risk probability. **B**. ROC curve of the logistic model. The receiver operating characteristic curve shows an AUC of 0.84, with the false positive rate (specificity) on the horizontal axis and the true positive rate (sensitivity) on the vertical axis

To enhance the interpretability and clinical usability of the prediction model, a standard multivariable logistic regression was constructed and a nomogram was developed for individualized risk estimation of GC, as illustrated in Fig. 2A. The nomogram incorporated five predictive variables: pre-CRP, gallbladder diameter, effusion, BA, and ALB. Each variable is assigned a score based on its contribution to the model. The cumulative score corresponds to a specific total point on the scale, which is then mapped to the estimated probability of GC. Among the predictors, elevated pre-CRP levels, increased gallbladder diameter, and the presence of effusion significantly contribute to higher total scores, thereby indicating a higher probability of GC. Although BA and ALB have lower weights in the final model, they provide complementary value by reflecting systemic immune and nutritional status. The inclusion of ALB—despite its lack of statistical significance in multivariate regression—supports the nomogram’s interpretability from a physiological perspective.

The model’s discriminative ability was further evaluated by receiver operating characteristic (ROC) analysis (Fig. 2B). The area under the ROC curve (AUC) was 0.84, indicating good overall predictive performance in distinguishing GC from non-GC patients. The ROC curve shows a balanced trade-off between sensitivity and specificity, particularly in clinically relevant threshold ranges. Additionally, the model achieved an accuracy of 84.35%, a precision of 82.66%, a recall (sensitivity) of 84.35%, and an F1-score of 0.82 on the independent test set. These results suggest that the model may be effectively applied in preoperative risk stratification to identify high-risk GC patients, enabling earlier surgical intervention and improved clinical outcomes.

### Comparison of multi-model prediction performance

To further explore the performance differences of various algorithms in the prediction of gangrenous cholecystitis, this study constructed nine mainstream machine learning models, including Random Forest, XGBoost, LightGBM, Gradient Boosting Decision Tree (GBDT), Support Vector Machine (SVM), Artificial Neural Network (ANN), K-Nearest Neighbour (KNN), Linear Discriminant Analysis (LDA), and Stacking ensemble model. The performance of each model was evaluated on an independent test set, with a comprehensive consideration of core metrics such as accuracy, AUC, F1-score, recall rate (Sensitivity), and specificity.

**Fig. 3:**
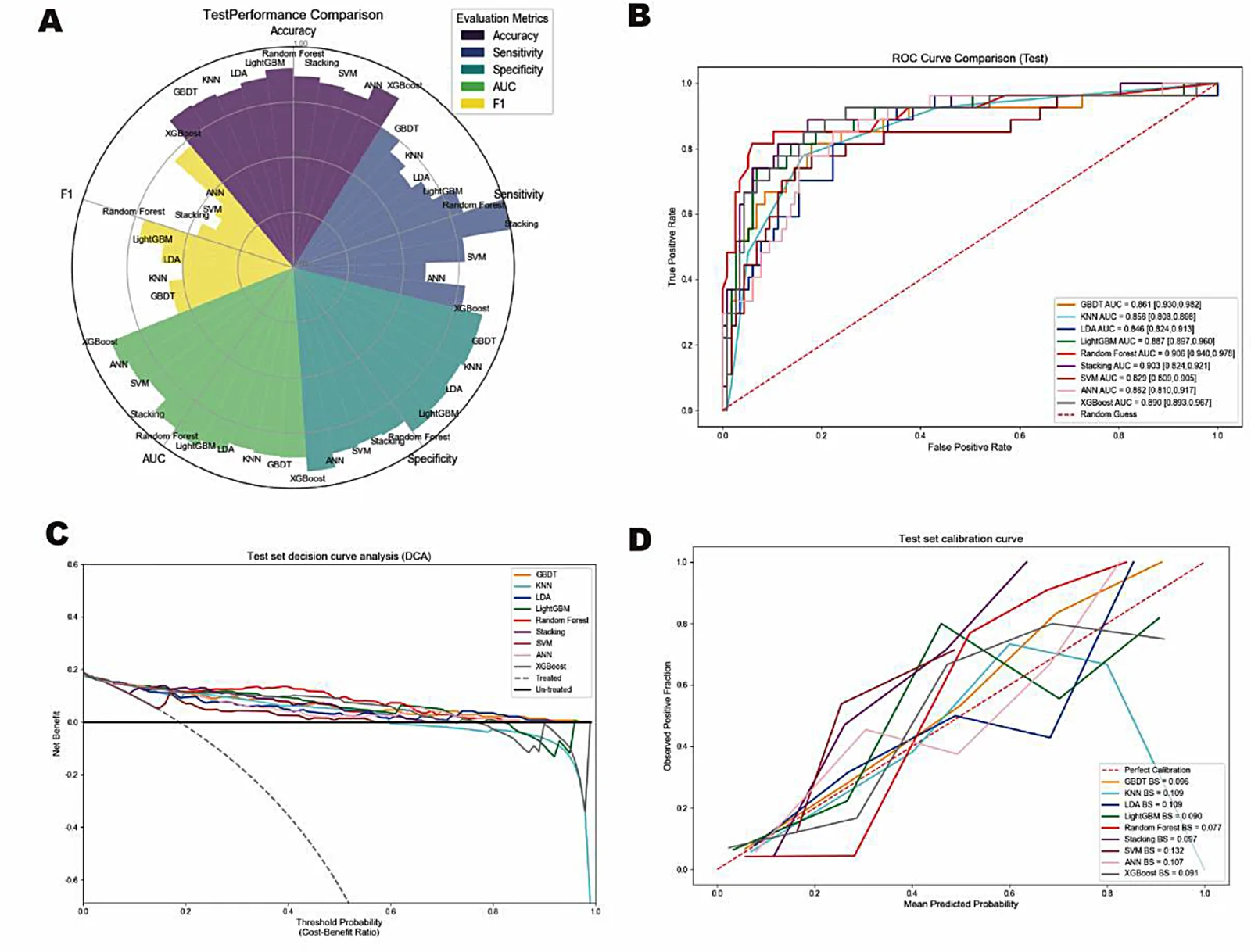
Comparison of prediction results from nine models. **A**. Radar chart of different models under various performance metrics. The figure shows the performance of nine models in terms of accuracy, AUC, F1-score, and recall rate. The larger the area, the better the overall performance. **B**. Comparison of ROC curves of different models on the test set.A larger area under the curve (AUC) indicates stronger model recognition capability. **C**. Clinical decision curve (DCA) analysis for each model. The larger the net benefit shown within the threshold interval, the stronger the clinical utility. **D**. Comparison of calibration curves for each model. Lower values indicate that the predicted probability is highly consistent with the actual results, indicating high model prediction reliability

As shown in the radar chart (Fig. 3A), XGBoost and Random Forest outperformed other models across most metrics, particularly in AUC and accuracy. These results reflect the strong non-linear modeling capabilities and adaptability to complex feature interactions inherent to ensemble-based learners. Conversely, traditional classifiers such as LDA and KNN performed relatively poorly in terms of sensitivity and F1-score, suggesting limited suitability for high-stakes clinical risk prediction. The Stacking model demonstrated relatively balanced performance across all dimensions, indicating good generalizability. Receiver operating characteristic (ROC) analysis further confirmed the superior discriminatory power of the ensemble models (Fig. 3B). XGBoost achieved the highest AUC (0.90), followed closely by Random Forest (0.89) and LightGBM (0.88). These curves were smooth and consistently elevated across thresholds, implying robust sensitivity-specificity trade-offs. While GBDT and Stacking also exhibited strong ROC profiles, shallow learners such as KNN and SVM underperformed, reflecting instability in their threshold-level decision boundaries.

Decision curve analysis (DCA), a method for assessing net clinical benefit across varying threshold probabilities, is illustrated in Fig. 3C. XGBoost, Random Forest, and Stacking models yielded the highest net benefits across a broad range of thresholds. Their performance surpassed both the “treat-all” and “treat-none” strategies, reinforcing their potential utility as preoperative decision-support tools for identifying high-risk GC patients. In terms of calibration performance (Fig. 3D), XGBoost, Random Forest, and Stacking models again stood out. Their predicted probabilities closely aligned with observed event rates, as reflected in the proximity of their calibration curves to the 45-degree reference line. In contrast, models such as KNN and SVM exhibited substantial deviations in moderate-to-high risk ranges, indicating suboptimal reliability for probability-based clinical decisions.

**Fig. 4:**
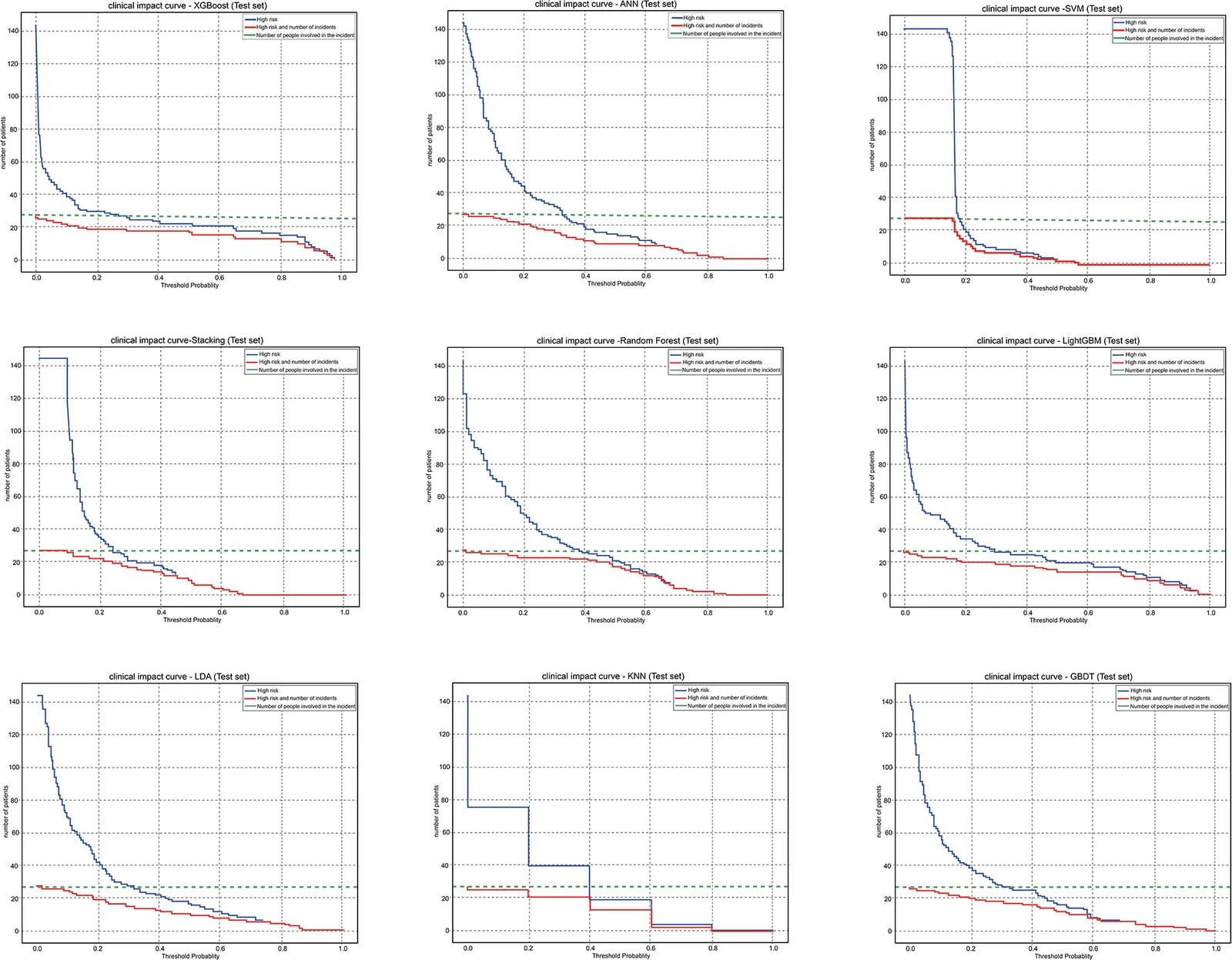
CIC of nine classification models on the test set. The horizontal axis represents the risk prediction probability threshold, and the vertical axis represents the number of samples. The blue curve indicates the number of patients predicted by the model as high-risk (positive), the red curve indicates the number of patients who were actually diagnosed with gangrenous cholecystitis (GC), and the green dashed line serves as the reference line for the total number of actual GC patients. The closer the red line is to the blue line, the lower the model’s false positive rate; the higher the overall position of the red line, the more effectively the model captures high-risk patients

In addition, the clinical prediction curves shown in Fig. 4 further reveal the differences in ranking stability among different models. XGBoost and Random Forest have the lowest prediction errors, the clearest classification boundaries, and the highest rankings. In contrast, KNN and SVM have obvious errors fluctuations and unstable performance. In terms of overall performance, stability, and statistical significance, XGBoost and Random Forest have the most prominent advantages in GC prediction and are suitable as recommended models for clinical decision support.

### Pairwise statistical comparison of model performance using DeLong’s test

**Fig. 5:**
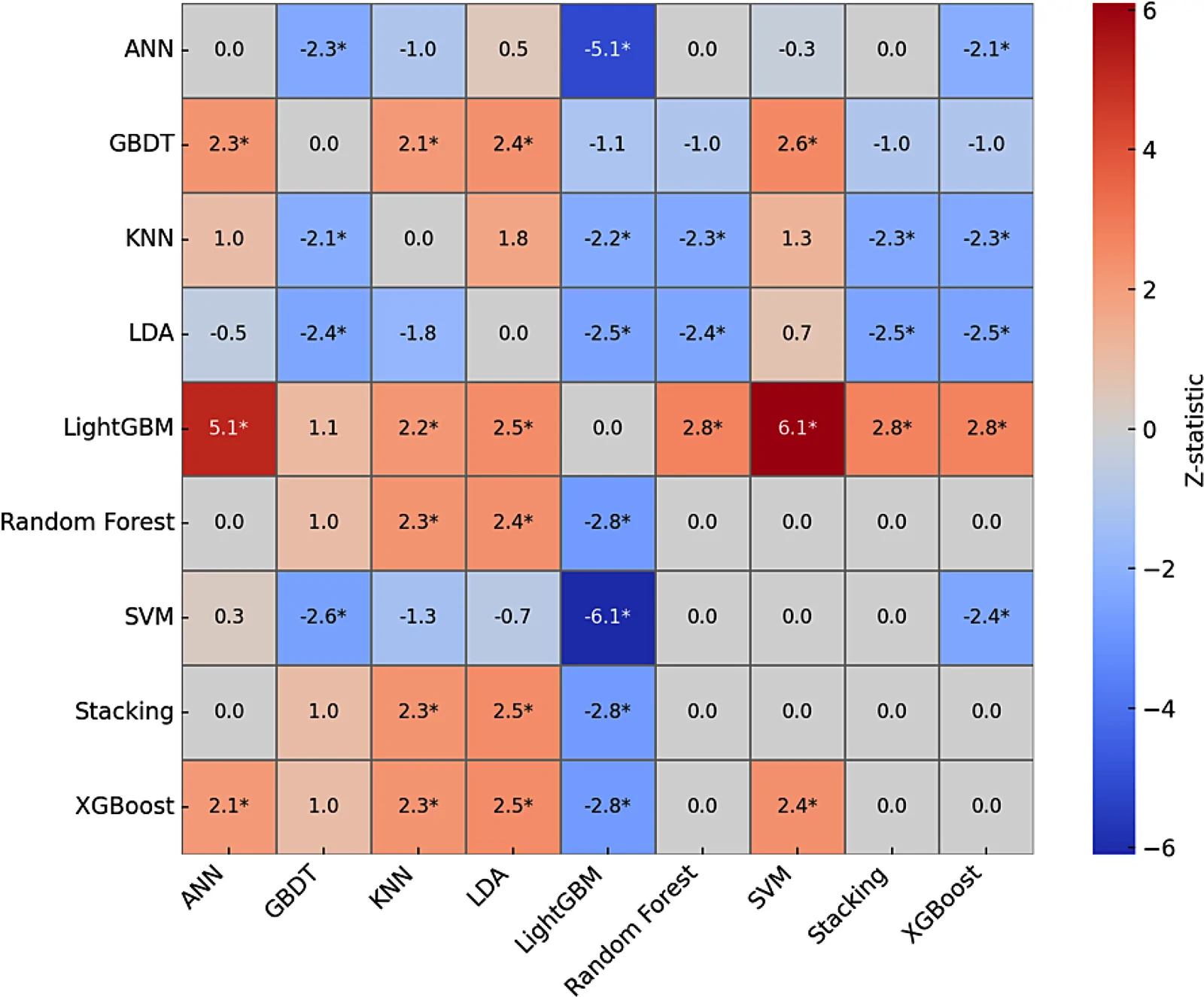
Heat map of AUC differences between nine prediction models. Differences in Z-test values between models. Each cell in the figure represents the Z-test statistic between two models. The deeper the red, the more significant the left model is than the model below it, and the opposite is true for blue. The larger the Z-value, the more significant the difference. The diagonal line represents self-comparison

To quantitatively evaluate the statistical significance of performance differences among the models, DeLong’s test was applied for pairwise comparison of AUC values on the test set. The Z-statistic matrix is presented in Fig. 5, where warmer colors (red) indicate significantly higher AUC values of the model on the y-axis compared to the one on the x-axis, while cooler colors (blue) represent the opposite. The results demonstrate that both XGBoost and Random Forest achieved AUC scores (AUC = 1.000)。Among the nine established models, XGBoost and Random Forest presented favorable overall performance. These two models exert important value in the preoperative differential diagnosis of gangrenous cholecystitis and may provide objective reference for clinicians to formulate individualized clinical decision-making.XGBoost showed statistically significant superiority over several other models, including KNN, SVM, ANN, and LDA, with |Z| values exceeding 2.0 in most comparisons, confirming meaningful differences in classification performance. Although Random Forest and XGBoost shared identical AUC values, nuanced differences were observed in secondary performance indicators. Random Forest displayed slightly greater stability in accuracy and specificity, suggesting its suitability in clinical scenarios where minimizing false positives is critical (e.g., surgical decision-making). In contrast, XGBoost exhibited marginally better recall (sensitivity = 0.54) and F1-score (F1 = 0.50), indicating a stronger potential for early detection where sensitivity is prioritized (e.g., preoperative triage or risk stratification).

On the other hand, traditional or shallow learners such as KNN, LDA, SVM, and ANN underperformed in this complex prediction task. Despite achieving moderate AUC values (e.g., ANN = 0.8475, LDA = 0.8441, SVM = 0.8366), these models failed to demonstrate competitive calibration or clinical utility in decision curve analysis (DCA). Their limited capacity to handle non-linear interactions and multi-source heterogeneity may explain their inferior performance in predicting GC, a condition with complex inflammatory and anatomical features. In summary, the DeLong analysis reinforces the earlier findings by confirming that ensemble-based algorithms. particularly XGBoost and Random Forest, not only yield superior raw performance metrics but also exhibit statistically significant advantages over classical methods, making them ideal candidates for clinical implementation.

### SHAP interpretability analysis

**Fig. 6:**
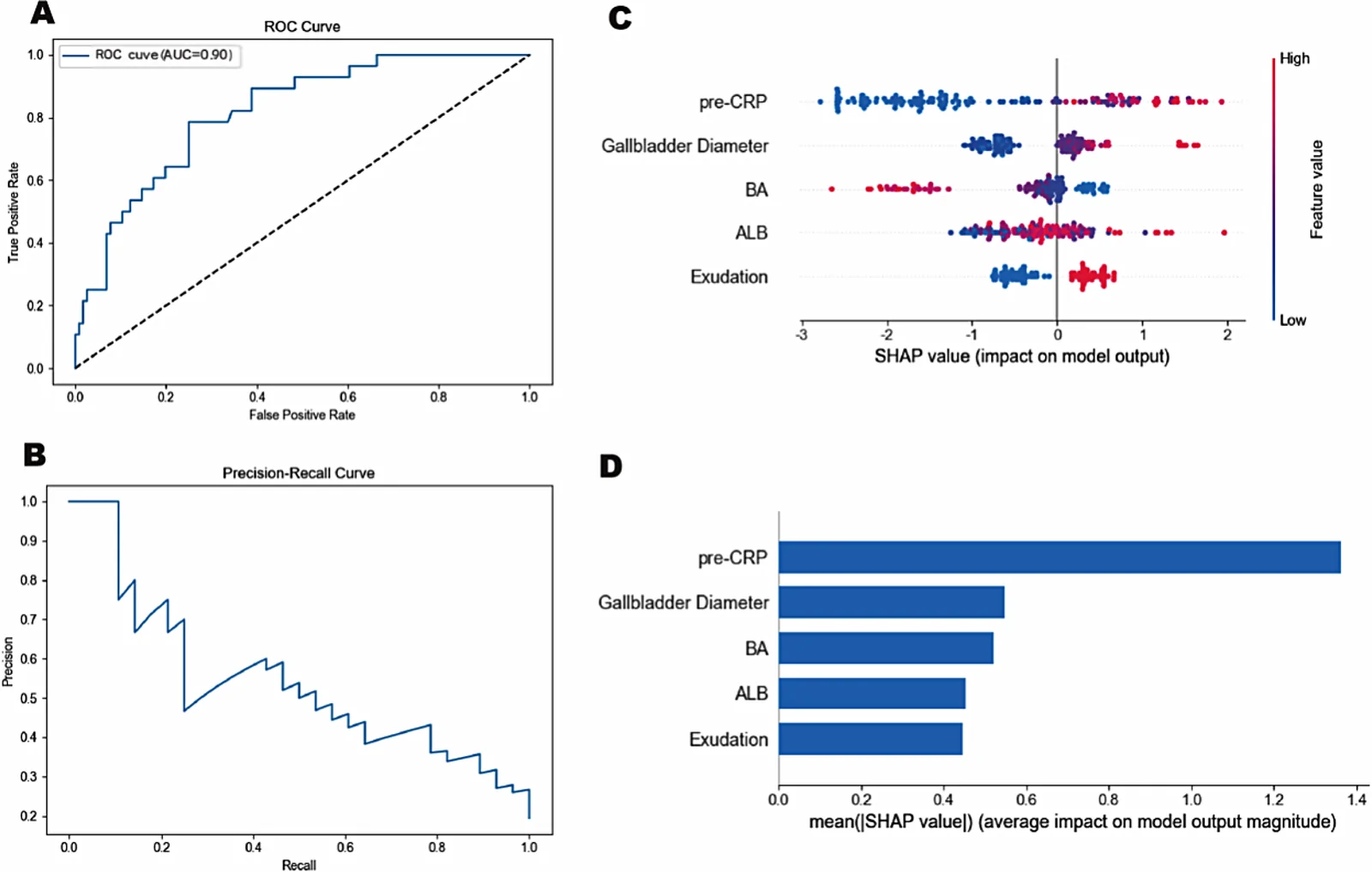
Performance evaluation and interpretability analysis of the XGBoost model for predicting GC. **A**. ROC curve of the XGBoost model, showing an AUC of 0.90, indicating good discriminative performance in distinguishing GC from non-GC patients. **B**. PR curve demonstrating that the model maintains relatively high precision across low-to-moderate recall levels, which is desirable for clinical screening tasks. **C** SHAP summary plot illustrating the contribution of each feature to the model’s output. Higher CRP and gallbladder diameter values were strongly associated with increased GC risk, while lower albumin levels and the presence of exudation also contributed positively to risk prediction. BA showed bidirectional effects, possibly reflecting underlying interactions. **D** Bar plot of mean absolute SHAP values, ranking feature importance in descending order. CRP emerged as the most influential predictor, followed by gallbladder diameter and BA

To further elucidate the decision-making process of machine learning models in predicting the risk of gGC and to enhance their clinical interpretability, SHAP (SHapley Additive exPlanations) analysis was performed on the two best-performing models: XGBoost and Random Forest. SHAP values quantify the marginal contribution of each feature to individual predictions and enable global interpretation of feature importance and directional impact on the output risk. As shown in Fig. 6, the XGBoost model was first evaluated and interpreted. The ROC curve (Fig. 6A) indicated an area under the curve (AUC) of 0.80, reflecting good overall discriminative performance in distinguishing GC from non-GC cases. Notably, the curve remained smooth in the high-sensitivity region, suggesting that the model is suitable for screening tasks where minimizing false negatives is critical. Complementarily, the precision-recall curve (Fig. 6B) demonstrated that the model maintained relatively high precision (0.4–0.7) when recall was below 0.6. However, precision dropped substantially as recall approached 1.0, indicating a trade-off in specificity at higher sensitivity levels. This behavior suggests that while the model is effective for identifying high-risk individuals, it may also misclassify some low-risk patients as high-risk, which is acceptable in settings prioritizing early detection. The SHAP summary plot (Fig. 6C and D) provides insight into individual feature contributions. CRP emerged as the most influential positive predictor, with higher values consistently associated with increased predicted risk. Conversely, ALB showed a negative association, implying that poor nutritional status is linked to elevated GC risk. Effusion, as a binary feature, had a clear risk-enhancing effect when present. Interestingly, gallbladder diameter and BA demonstrated variable impacts depending on their values, suggesting potential nonlinear or interaction effects among predictors.

**Fig. 7:**
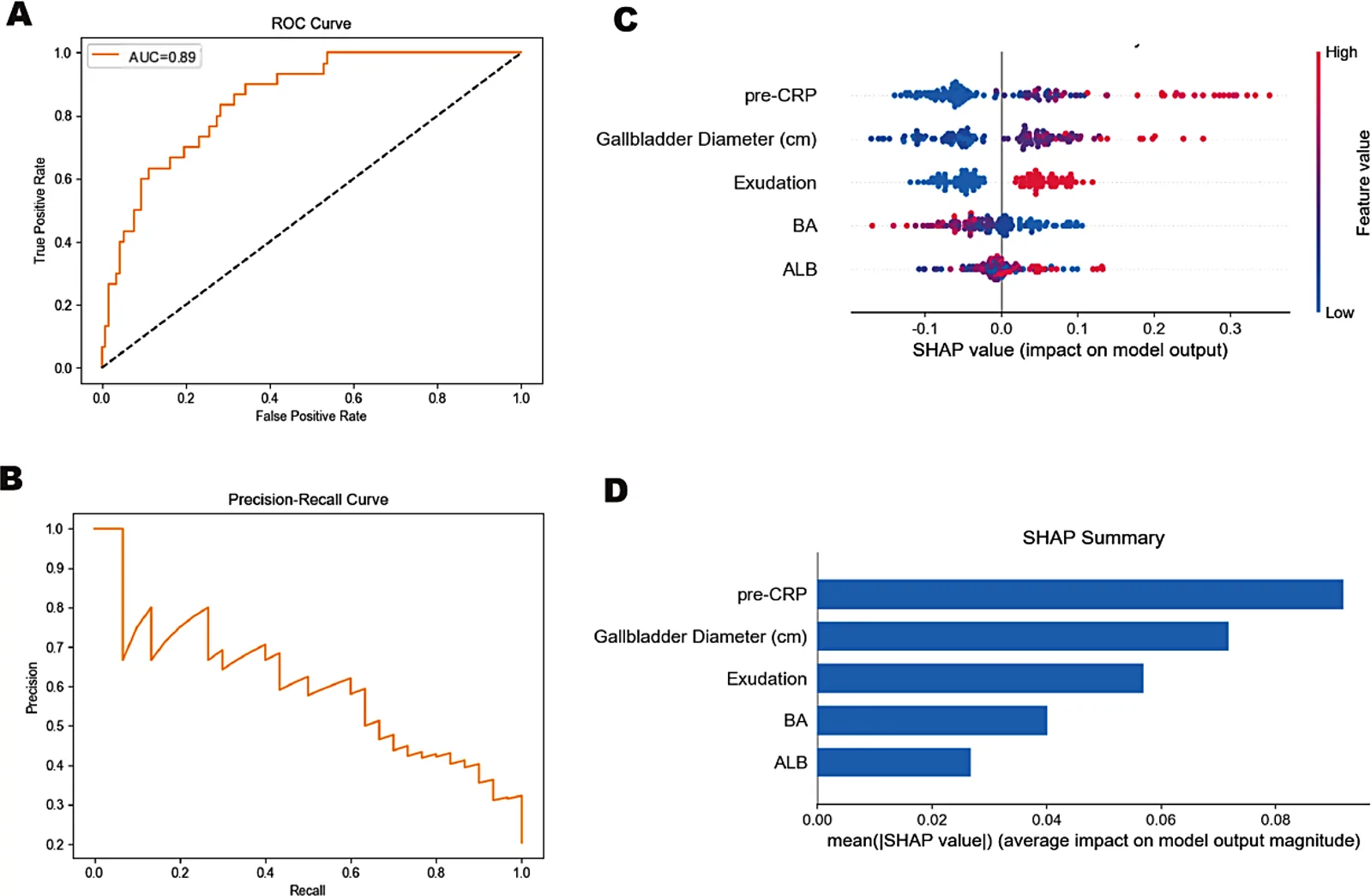
Performance evaluation and SHAP-based interpretability analysis of the Random Forest model. **A**. ROC curve of the Random Forest model showing an AUC of 0.89. **B**. PR curve demonstrating that the model maintains moderate to high precision (> 0.5) at recall levels up to 0.5. **C**. SHAP summary plot illustrating the contribution of each feature to the model’s output for individual predictions. **D**. SHAP bar chart showing the mean absolute SHAP values, quantifying the average importance of each feature

Given its comparable performance to XGBoost, the Random Forest model was further analyzed in terms of predictive performance and feature interpretability. As shown in Fig. 7A, the ROC curve for the Random Forest model yielded an AUC of 0.89, outperforming the baseline logistic regression model. The curve maintained a smooth and convex profile, particularly in the low false positive rate region, indicating the model’s strong early detection capability and robustness in distinguishing GC patients at lower error thresholds The precision-recall curve (Fig. 7B) revealed that the model retained good predictive precision (≥ 0.5) across low-to-moderate recall ranges (0.1–0.5), suggesting suitability for scenarios requiring a balanced trade-off between sensitivity and specificity. This balance is especially valuable in clinical settings aiming to avoid both missed diagnoses and excessive false alarms. The SHAP summary plot (Fig. 7C and C) showed that CRP and gallbladder diameter remained the most influential positive predictors, exhibiting a consistently right-skewed distribution of SHAP values. This indicates their dominant and directionally stable contributions across most individual cases. Effusion, a binary imaging variable, also emerged as a strong categorical discriminator, reaffirming its diagnostic utility in GC risk stratification. In contrast, BA exhibited predominantly negative SHAP values, implying an inverse relationship with GC risk in some patient subgroups. Albumin (ALB) demonstrated a more dispersed SHAP pattern, suggesting sample-dependent effects and potential interactions with other variables, reflecting its multifactorial role in systemic inflammation and nutritional status.

Collectively, the Random Forest model not only achieved strong discriminative power but also offered high interpretability through SHAP analysis. This dual advantage makes it well-suited for clinical decision-support contexts where both predictive accuracy and model transparency are essential.

### Model evaluation strategy

This study strictly followed the analytical workflow: dataset partitioning → feature selection on the training set → model training → validation on the test set. All feature selection procedures, including the t-test and LASSO regression, were conducted exclusively on the training set.It should be noted that all performance metrics, including area under the curve (AUC), accuracy, and F1-score, were calculated using the independent internal test set, which remained fully independent and was used for the final validation of the model. The training set was solely employed for model construction and hyperparameter optimization, and its results were not reported as the final predictive performance. No resampling data was used for the evaluation of outcome performance.

## Discussion

At present, predictive models for gangrenous cholecystitis primarily rely on traditional logistic regression models or empirical scoring systems. While these GC risk scoring methods are simple and practical, they are limited by fixed thresholds and static variables, making them ill-suited to the clinical heterogeneity observed among patients. Therefore, this study utilised single-centre clinical retrospective data to develop a preoperative predictive model for gangrenous cholecystitis that integrates multiple risk factors and offers high interpretability. The study systematically screened key variables, compared the performance of various mainstream machine learning models, and employed the SHAP method to enhance model transparency and credibility, thereby providing an intelligent decision-support tool for the early identification of high-risk GC patients prior to surgery.

Compared to traditional models such as logistic regression or LDA, XGBoost offers notable advantages in capturing nonlinear relationships and handling feature interactions without requiring extensive feature engineering [15, 16]. Furthermore, its robustness against multicollinearity and ability to perform implicit feature selection improve model stability and generalizability, which are often limitations in linear or shallow learning methods. In our multi-model comparison, while Random Forest also achieved high performance, XGBoost demonstrated better recall and F1-score, indicating superior capability in capturing subtle patterns associated with GC risk. From a clinical perspective, the features identified as most important by SHAP analysis align well with current pathophysiological understanding of GC, enhancing the model’s interpretability. CRP emerged as the most influential predictor, reflecting the critical role of systemic inflammation in the development of gangrenous changes [17, 18]. The positive correlation between higher CRP levels and GC risk reinforces previous findings and provides a reliable biochemical signal for early clinical alert [18, 19].

Gallbladder diameter, another key variable, reflects progressive distension and tension within the gallbladder wall, often indicating advanced inflammation or ischemic injury [2, 14, 20]. Its strong positive impact on model output, as quantified by SHAP values, underscores its imaging diagnostic value. In contrast, serum albumin (ALB) showed a predominantly negative contribution, suggesting that hypoalbuminemia, potentially reflecting poor nutritional status or systemic catabolic state, may serve as an indirect marker of vulnerability to severe inflammatory damage [21–23]. Interestingly, This study identified the basophil (BA) percentage as a key predictive factor. However, basophils do not exert a dominant role in acute surgical inflammation. Accordingly, BA percentage may merely serve as a potential surrogate marker of immune dysregulation or a dataset-specific discriminative variable. Therefore, no definite causal relationship was established between BA percentage and the progression from acute cholecystitis to gangrenous cholecystitis. The interpretation of basophil percentage (BA) should be regarded as hypothesis-generating rather than a definitive mechanistic conclusion. The presence of pericholecystic exudation, a binary imaging feature, was also consistently associated with increased GC risk, confirming its established role in reflecting local disease severity [24, 25].

Compared with the prediction models applied in existing studies, the multi-model ensemble framework constructed in this study demonstrates significant advantages in terms of accuracy, generalisation ability, and interpretability. Okuda et al. proposed a deep learning model based on convolutional neural networks (CNNs) for GC detection in CT images, achieving an AUC of 0.84, which was significantly better than the performance of three experienced radiologists (AUC 0.63) [12]. However, this model heavily relies on high-quality image data and lacks integration of clinical variables, limiting its generalisability in scenarios where non-image data is unavailable. Karthikarajam et al. developed a logistic regression model using demographic and laboratory indicators to screen for high-risk factors for GC, such as elevated white blood cell count (WBC), alkaline phosphatase (ALP), and serum glutamic-oxaloacetic transaminase (SGOT/SGPT), as well as thickened gallbladder walls [10]. However, the model’s predictive performance was not validated on an independent validation set, and the model’s calibration and clinical decision curves were not evaluated. In contrast, this study comprehensively considered preoperative imaging, inflammatory, nutritional, and immune markers and systematically compared the multidimensional performance of nine mainstream machine learning models.

The findings of the present study are primarily applicable to our institution. For patients presenting with acute cholecystitis, those identified as high-risk for gangrenous cholecystitis through model assessment may be prioritized for early surgical intervention. This approach not only minimizes the risk of severe infection and mitigates systemic damage induced by exacerbated inflammation but also enhances perioperative vigilance among clinicians, prompting more intensive monitoring of such patients.

Furthermore, this model may serve as a valuable early warning tool for primary care facilities. Patients stratified as high-risk for gangrenous cholecystitis can be promptly referred to upper-level hepatobiliary surgery centers for definitive surgical management, which may reduce patient waiting time and diminish the risk of severe complications.

In summary, the predictive models established in this study are well-suited for high-risk screening in emergency general surgery, helping to avoid critical adverse events such as intraoperative gallbladder perforation, intra-abdominal infection, and sepsis. Meanwhile, the Random Forest model, characterized by superior specificity and stability, is suitable for refined risk stratification of patients undergoing elective surgery, thereby facilitating the avoidance of unnecessary overtreatment.

This study has several limitations. As a single-center retrospective investigation with a limited sample size and a small number of gangrenous cholecystitis cases, potential selection bias and model overfitting cannot be fully excluded. In addition, the current discriminative performance was validated only through internal evaluation at a single institution. Further multicenter prospective cohort studies with larger sample sizes are required for external validation, to improve the generalizability and robustness of the model and obtain more universal predictive outcomes. Such efforts will facilitate the clinical translation and practical application of machine learning-based predictive models in hepatobiliary surgery.

## Data Availability

The datasets generated and analyzed during the current study are available from the corresponding author on reasonable request.
